# Adopting Virtual Visits for Parkinson's Disease Patients During the COVID-19 Pandemic in a Developing Country

**DOI:** 10.3389/fneur.2020.582613

**Published:** 2020-10-27

**Authors:** Ali Shalash, Mai Fathy, Noha L. Dawood, Eman Hamid

**Affiliations:** Department of Neurology, Faculty of Medicine, Ain Shams University, Cairo, Egypt

**Keywords:** Parkinson's disease, telemedicine, COVID-19, virtual visits, feasability

## Abstract

**Objective:** Telemedicine has been increasingly used, especially during the COVID-19 pandemic; however, limited data are available from developing countries. The present study aimed to evaluate the feasibility, satisfaction of patients and physicians, and quality of service provided during virtual visits for Parkinson's disease (PD) patients during the COVID-19 pandemic and the associated limitations.

**Methods:** Thirty-nine PD patients were contacted to schedule virtual visits using the Zoom application. Thereafter, we rated the feasibility, satisfaction, and quality of service provided by virtual visits using patients' and physicians' questionnaires.

**Results:** Twenty-one out of 39 PD patients were scheduled for virtual visits. Nineteen virtual visits out of 21 (90.5%) were conducted successfully; 16 of these were accomplished in the first attempt (76.2%). The scores of satisfaction, quality of service, and set-up/preparation were 9.5 (8.5–10), 9.5 (9–10), and 8 (5–10) for the patients and 9 (7–10), 8 (6–10), and 10 (10–10) for the physicians, respectively. The average time that was saved was 270.79 ± 142.17 min, while an average of 76.38 ± 95.15 km of travel was avoided for the patients per visit. The most common limitations for conducting virtual visits were a lack of Internet connection and the inability to use technology (75%).

**Conclusions:** The present study showed the feasibility and the high satisfaction level of patients and physicians as well as the favorable service quality of virtual visits for PD in a developing country during the COVID-19 pandemic. However, the lack of Internet connectivity and the inability to use technology were the main limitations.

## Introduction

Parkinson's disease (PD) is the second most common neurodegenerative disease, and the healthcare burden of this disease is increasing across the world. Recently, telemedicine has been increasingly adopted for managing PD patients, owing to the rising burden, limited availability of specialists, recent evidence of its applicability, and advances in technology (1, 2).

Furthermore, healthcare services for chronically ill patients, including PD patients, were compromised during the COVID-19 pandemic, owing to the lockdown and the direction of healthcare resources toward its containment. Moreover, impaired mental health, compromised physical activity, and poorer quality of life of PD patients have been reported during the COVID-19 pandemic, implying the importance of continuing their care, especially *via* telemedicine (3). The current situation has led to the use of telemedicine by physicians as an alternative to in-person visits.

Several studies have demonstrated the feasibility, time-saving ability, cost-efficiency, high satisfaction level, and comparable outcomes of virtual visits and in-person visits (4–7). However, these studies have reported major limitations, including incomplete neurological examination, reimbursement of physicians, the inability of older and less educated patients to use it, and technological obstacles, especially those with limited access to optimum Internet services (2). However, all these reports were from studies performed in developed countries. In developing countries, cultural acceptance and awareness might be greater barriers than technical issues for the adoption of telemedicine (8, 9). Therefore, further research is needed on different populations to identify the barriers for the adoption of telemedicine.

The present study was designed with the aim of evaluating the feasibility and the satisfaction level of patients and physicians as well as the quality of service and the limitations involved in virtual visits for PD patients during the COVID-19 pandemic.

## Methods

Fifty-one PD patients who were regularly followed up in the movement disorders clinic, Ain Shams hospitals, Cairo, with available data and recent comprehensive assessment in a previous in-person visit from August 2019 to February 2020 (within 6 months before the COVID-19 pandemic-related lockdown in Egypt) were identified from our data registry. Available data and assessments included the contact information, demographic data, socioeconomic status, education years, motor assessment using Movement Disorders Society—Unified Parkinson's Disease Rating Scale and Hoehn and Yahr Scale, and cognitive evaluation using the Mini-Mental State Examination (MMSE). The patients were previously diagnosed with PD as per the MDS diagnostic criteria (10). Patients with atypical or secondary Parkinsonism and those who were unable to complete the questionnaire, such as those with severe cognitive impairment, were excluded. Thirty-nine out of the 51 patients could be reached *via* telephone and were asked whether they were interested in and capable of participating in virtual follow-up visits; they were then invited for a free one-time virtual visit.

Telemedicine sessions were performed by the movement disorders experts during May and June 2020, using the Zoom application. Before the visits, test sessions were offered and performed when required by the patients or their caregivers; furthermore, instructions for a proper virtual visit were discussed, such as the use of a device with a high-resolution camera, good lighting, the help of another person, and avoiding overexposure. The meeting link and prescribed medications were sent to the patients *via* WhatsApp messages.

During the virtual visits, the patients were assessed for motor and non-motor symptoms, medications, physical activity, and the impact of the COVID-19 pandemic. The motor examination was conducted during the virtual visit, including finger tapping, hand movements, and leg agility for bradykinesia, tremor assessment (during rest, posture, and action), and arising from chairs and gait, while rigidity and postural instability could not be examined virtually. The feasibility of virtual visits was calculated as the ratio of successful visits to that of the scheduled visits (5, 6).

Following the virtual visits, the patients were asked *via* phone calls about the quality of service, visit set-up/preparation, and their satisfaction using a patient questionnaire (16 questions) developed by Hanson and colleagues, (6) in addition to two questions about the use of telemedicine during the COVID-19 pandemic. The calls were made by physicians other than the interviewing physicians within 2 days from the virtual visit. The patient questionnaire was translated into Arabic and validated as per standard methods. In a similar manner, the interviewing physician rated similar visit outcomes using the physician questionnaire (11 questions) developed by Hanson and colleagues (6). Both questionnaires include questions with a 10-point Likert scale, with 1 = highly disagree or least satisfied and 10 = highly agree or most satisfied for a comparison of the virtual and previous in-person visits regarding the following three domains: service quality, visit set-up/preparation, and patients'/physicians' satisfaction (6). Furthermore, the patients were asked about the cost and the distance involved in actual hospital visits.

All the patients provided their consent for study participation; the study was approved by the ethical committee of the Faculty of Medicine, Ain Shams University, and conformed to the ethical standards of the Declaration of Helsinki.

### Statistical Analyses

Data were analyzed using SSPS®, version 20.0 (IBM, San Francisco, CA, USA). The data were described as frequency and percentage values for qualitative data and mean and standard deviation or median and range values for quantitative data. Chi-square test was used to compare the non-parametric variables, while *t*-test was used to compare the parametric variables between patients who declined virtual visits and those for whom virtual visits were performed successfully.

## Results

**Figure 1:**
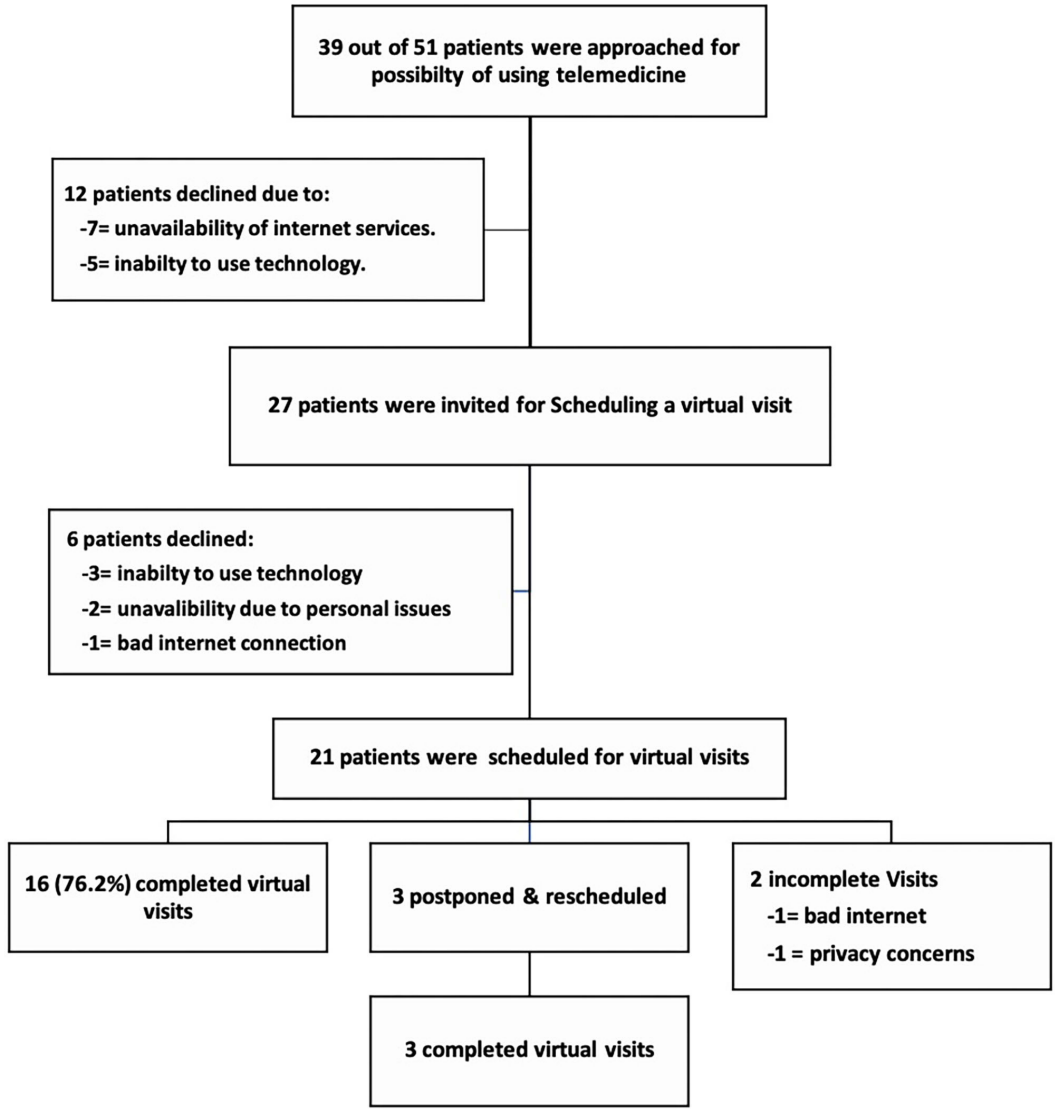
Flow chart of the invitation, enrollment, and performance of virtual visits for patients with Parkinson's disease during the COVID-19 pandemic.

Twenty-seven of the 39 patients (69.2%) expressed their interest and capability to participate in virtual visits, while 12 refused owing to the lack of Internet connection (seven patients) or inability to use technology (five patients). The aforementioned 27 patients were invited to schedule an online virtual visit. Twenty-one of the 27 patients were scheduled for virtual visits, while six patients apologized for the following reasons: bad Internet connection (one patient), inability to use the application (three patients), and unavailability due to personal issues (two patients) (Figure 1).

Sixteen of the 21 virtual visits were completed from the first appointment (76.2%); three virtual visits were postponed, then rescheduled, and completed. Thus, 19 visits (90.5%) were successfully performed at the patients' homes, while two visits could not be conducted; one was not feasible owing to Internet disconnection and another owing to privacy concerns (Figure 1). Thus, 48.7% of the approached patients (19 out of 39) could participate in the virtual visits, while the feasibility of the conducted virtual visits was 90.5% (including rescheduled visits) and 76.2% from the first attempt.

All the patients used smartphones and were assisted by a family member, while the doctors used their laptops. Test sessions were carried out successfully for five patients (26.3%) and followed by virtual visits.

### Barriers in Patient Enrollment and Virtual Visits

Virtual visits could not be performed for 20 of the 39 invited patients (51.3%). The main causes were absent or unstable Internet connection (nine patients, 35%) and the inability to use technology without caregiver support (eight patients, 40%). One patient declined owing to privacy concerns (5%), while two patients withdrew owing to personal issues. There were no significant differences in age (*p* = 0.757), sex (*p* = 0.888), education years (*p* = 0.226), residence (*p* = 0.716), functioning (*p* = 0.462), and MMSE (*p* = 0.359) of patients who declined visits owing to Internet and technology usage problems (17 patients) and those for whom virtual visits were performed successfully (19 patients).

### Patients' and Physicians' Satisfaction

All patients for whom virtual visits were initiated were able to complete the visit and the questionnaire. The median scores for satisfaction, quality of service, and set-up/preparation of patients were 9.5 (8.5–10), 9.5 (9–10), and 8 (5–10), respectively (Table 1). Twelve patients established a personal connection with the physicians; however, this percentage was lower than that for in-person visits (63.1%); four patients reported a similar personal connection (21%), while three did not establish a personal connection (15.9%). Most patients were highly satisfied (15, 78.9%) or satisfied (three, 15.9%) with the recommendations.

**Table 1:** Demographic and clinical characteristics of patients and outcomes of the virtual visits.

	**Mean/No/Median**	**SD/frequency**	**Range**
Age	56.0	10.51	31–72
Sex: male/female	13/6	68.4/31.6%	
Socioeconomic status	Low: 11 (57.89%), middle: 8 (42.10%), High: 0		
Highest level of education	Illiterate	5 (26.3%)	
	Read and write	2 (10.5)	
	Preparatory school	3 (15.8%)	
	High school	4 (21.1%)	
	University	5 (26.3%)	
Years of education	8.368	6.3614	0–18
Age of onset	50.158	11.3285	25–62
Duration of illness	5.605	3.5652	0.5–14
MDS UPDRS-III-OFF^a^	42.294	20.2632	10.0–71.0
Hoehn and Yahr Scale^a^	2.444	0.8726	1.0–4.0
MMSE-total^a^	27.588	2.3994	23.0–30.0
Duration of virtual visit (min)	27.778	7.01	20.0–44.0
**Patients' outcome**			
Patients' satisfaction (median Q7, 8, 9, 17, 18, 19)	9.5		8.5–10
Patients' set-up/preparation (median Q5)	8.0		5–10
Patients' quality of service (median Q10, 11, 12, 13)	9.5		9–10
Saved costs for patients (Egyptian pounds)	163.95	204.98	15–800
Saved time for patients (transportation–waiting) (min)	270.79	142.17	105–540
Saved kilometers for patients	76.38	95.15	5.2–318
**COVID-related questions**			
Favoring future telemedicine in general or during pandemic	Yes, all the time		14 (73.7%)
	Only in COVID-19		5 (26.3%)
	No, I do not want		0
Do you think that telemedicine sessions can be an alternative for healthcare services during the COVID-19 pandemic?	10		5–10
**Physicians' outcome**			
Physicians' satisfaction (median Q14,15,16)	9		7- 10
Physicians' set-up/preparation (median Q5)	10		10–10
Physicians' quality of service (median Q7,8,9, 10, 11, 12, 13)	8		6–10
Saved time for physicians (transportation)	90	0.0	90–90
Saved kilometers for physicians	40.84	18.26	20–56

*MDS-UPDRS, MDS-unified Parkinson's disease rating scale; MMSE, mini-mental state examination*.

a *Conducted in the last in-person visit before the pandemic lockdown*.

Furthermore, the patients were very likely (17, 89.5%) or likely (two, 10.5%) to recommend telemedicine to other patients. Most patients (14, 73.7%) expressed a desire to use virtual visits beyond the pandemic, while five patients (26.3%) were interested in virtual visits only during the pandemic. All patients, except one, reported that virtual visits could be a satisfactory alternative to in-person visits during the pandemic (Supplementary Table 1).

**Figure 2:**
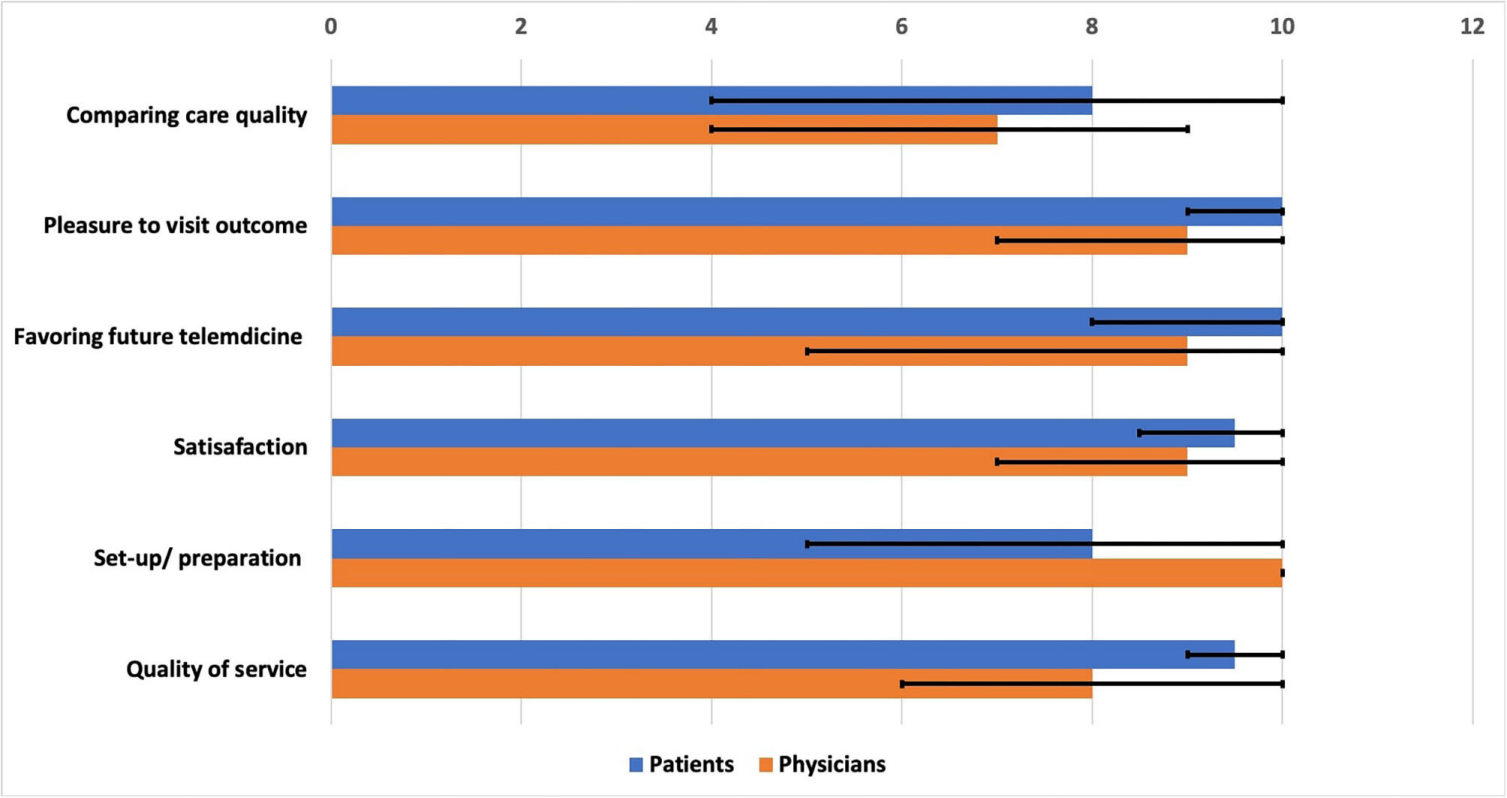
Satisfaction, set-up, preparation, and service quality of virtual visits for Parkinson's disease patients as rated by the patients and the physicians. The chart describes the median and the ranges of different items.

Regarding physicians, the scores of satisfaction, quality of service, and set-up/preparation were 9 (7–10), 8 (6–10), and 10 (10–10), respectively. They highly preferred future telemedicine visits when possible (eight, 7–9) (Supplementary Table 2). The patients reported a comparable overall care of virtual visits compared to in-person visits (eight, 4–10) more than the physicians (seven, 4–9). Both the patients and the physicians were highly pleased with the outcomes of the virtual visits (10 and nine, respectively) (Figure 2).

### Time- and Cost-Saving

The average time of the visit set-up was 12.9 ± 14.3 for patients and 6 ± 3.55 for physicians. On average, 270.79 ± 142.17 min was saved, while 76.38 ± 95.15 km of travel for patients per visit was prevented. Furthermore, the average reduction in transportation costs was 163.95 ± 204.98 Egyptian pounds for patients per visit.

### Visit Assessments and Recommendations

The major motor complaints of the patients were bradykinesia/rigidity (63.2%), tremor (47.4%), and gait problems (57.9%), while the reported non-motor complaints included depression/anxiety (47.4%), constipation (36.8%), and sleep disturbance (31.6%). Bradykinesia and gait were assessed in all patients, while tremor and dyskinesia were observed in nine (47.4%) and five (26.3%) patients, respectively. The most common actions were the promotion of protective measures for COVID-19 (100%), adjustment of medication (94.7%), and promotion of physical activity during lockdown (84.2%).

## Discussion

The current study demonstrated the feasibility, high satisfaction level of patients and physicians, cost and time saving, and comparable quality of service of virtual visits for PD patients (as well as barriers of using telemedicine) at home in a developing country during a special situation. Moreover, the study identified the barriers of using telemedicine for PD in developing countries. To our knowledge, this is the first study on virtual visits for PD from developing countries during the COVID-19 pandemic.

The patients reported high satisfaction and comparable care of virtual visits, consistent with previous studies (4–7). However, lower feasibility and satisfaction scores were detected in the present study compared to the previous studies that could be attributed to bad Internet connectivity, poor quality of the patients' devices, and lower education and socioeconomic levels of the participants. Furthermore, lower physician satisfaction could be attributed to the interrupted Internet connection, poor resolution of some patients' devices, and less exposure from the patients' side.

It is noteworthy that most of our patients reported less personal connection with physicians than during in-person visits, in contrast to prior studies. Hanson and colleagues showed that 69.2% established similar personal connections with the interviewing physicians (6). A recent randomized controlled study reported no significant difference in the patients' satisfaction related to communication in the virtual and in-person visits (11). Low personal communication could be explained by technical problems, low quality of the image from the patients' side, and cultural perceptions. Cultural issues, including the patients' acceptance and resistance, are common barriers in telemedicine in developing countries, including Egypt (9, 12).

It is noteworthy that about half of our patients (52.6%) were illiterate or had a low education level in contrast to prior studies that mainly included highly educated and technologically competent patients. However, virtual visits had been conducted successfully through the support of an educated relative, denoting the possibility of reaching out to these patients *via* easily used and widely available online applications. The patients showed lower set-up and preparation scores than the physicians and that in previous studies; this could be due to the lower education level, unfamiliarity with technology, and lack of training in the application used.

Several studies used smartphones for remote assessment and monitoring of PD patients at home (1, 2). Moreover, virtual visits were conducted using smartphones in about 50% of the patients in previous studies (7). The current study showed 100% use of smartphones, implying more availability of these devices among patients belonging to different socioeconomic levels. The application (Zoom) currently used was easily used by the patients with a short time of setting up; however, delayed audio connection at the time of logging in was a common technical challenge.

The current study showed time and cost saving similar to that in previous studies (2, 5). Despite less money being saved as compared to that in other studies, due to the depreciation of the local currency and the fact that most patients lived in the same governorate (68.4%), the money saved was substantial for the patients, given their low socioeconomic level.

Virtual visits enabled the physicians to provide healthcare to PD patients, including comprehensive assessment, adjustment of medications, and delivery of recommendations. This study included ideal aspects of telemedicine, such as easy-to-use technology, combined use of synchronous and asynchronous programs, and previous in-person visits. However, other aspects, such as the use of high-resolution devices, support by technology experts, and high-speed Internet connectivity, were inadequate (2, 5).

Lack of awareness was described as a major barrier for adopting telemedicine in developing Middle Eastern countries, resulting in resistance from the doctors and the patients, in addition to poor infrastructure, lack of funding, and poor technological training (8, 9). In contrast, this study demonstrated the high feasibility of scheduled visits and acceptance of virtual visits among the participants. Moreover, most patients favored its use beyond the pandemic, while technical limitations were the main barrier. Moreover, it showed that social support could facilitate the use of telemedicine for patients with poor education level. However, further studies are required to confirm these findings in actual clinical practice.

Despite the high feasibility of scheduled virtual visits in the current study, about half (51.3%) of the approached patients declined the use of telemedicine virtual visits. The absence of or bad Internet connection and the inability to use technology were the main barriers, representing 85% of the declined invitations. These barriers, in addition to cultural resistance and lack of regulations, have been variably reported among Middle Eastern countries and other developing countries (9, 13). Consistently, most PD patients who participated in the virtual visit studies in developed countries were well-educated and more familiar with the technology (14). Furthermore, older patients with chronic illnesses have less access to the Internet (2). However, patients who did and did not participate in the visits showed similar cognition, education level, and age, emphasizing the impact of Internet availability and social support in technology use. Privacy concern was reported by a female patient; this is related to cultural and religious beliefs as a relevant obstacle for telemedicine in our region (9).

Overcoming the barriers of adopting telemedicine in developing countries is crucial for better healthcare of PD patients. Providing good Internet services and advanced infrastructure of communication technology to underserved as well as rural areas and support patients for good access to the Internet is necessary. The use of simple systems for telehealth services, patients' training, and the availability of well-educated family members might help overcome the technology-related obstacles (9). Furthermore, establishing satellite clinics in underserved and rural areas with a good Internet connection is another way to overcome the lack of Internet and technological problems at homes and increase access to telehealth services (15). These strategies could be implemented through a comprehensive telemedicine program for developing countries that use well-organized services, apply simple technology, adopt patient and healthcare provider training, and receive continuous funding (13). Moreover, promoting public awareness about the benefits of telemedicine is important for reforming the social perception of telemedicine, especially in developing countries (9). The COVID-19 pandemic and the related challenges emphasize the importance of establishing well-organized telehealth services all over the world, especially for chronic neurological diseases.

The relatively small study population was a major study limitation that could be attributed to the inclusion of only patients with available data and recent comprehensive assessment in the past 6 months before the lockdown and the mitigation of the lockdown restrictions in Egypt by the end of June 2020. Another limitation was the application of less organized telemedicine services, owing to the unusual emerging situation. However, this represents a real-life situation and more practical use of telemedicine. The current findings should be interpreted in the context of the unusual situation of inaccessibility of in-person health services that might impact the patients' acceptance of virtual visits. Therefore, further studies are warranted to investigate the use of telemedicine for PD patients in developing countries with a larger number of patients and longer follow-up periods during usual clinical practice.

## Conclusion

The current study describes the high feasibility, high satisfaction level, and favorable quality of service of virtual visits, implying the possibility of the rapid adoption of telemedicine in emerging situations and in developing countries. Furthermore, it identifies the barriers against telemedicine use in a developing country, especially poor Internet service and technical challenges. Stable Internet connectivity, use of simple technology, promotion of public awareness, availability of well-organized services, formulation of regulations, and reimbursement are required for the long-term establishment of effective telemedicine services.

## Data Availability Statement

The raw data supporting the conclusions of this article will be made available by the authors, without undue reservation.

## Ethics Statement

The studies involving human participants were reviewed and approved by The ethical committee of the faculty of medicine, Ain Shams University. The patients/participants provided their written informed consent to participate in this study.

## Author Contributions

AS contributed to the idea and conception, study design, data collection, statistical design and execution, writing of the first draft, and review and critique of the manuscript. MF and ND contributed to the research project execution, data collection, statistical analysis design and execution, writing and review of the first draft of the manuscript, and review and critique of the manuscript. EH contributed to the study design, research project execution, data collection, statistical analysis design and execution, writing of the first draft of the manuscript, and review and critique of the manuscript. All authors contributed to the article and approved the submitted version.

## Conflict of Interest

The authors declare that the research was conducted in the absence of any commercial or financial relationships that could be construed as a potential conflict of interest.
