# Supplementary material for: Adopting Virtual Visits for Parkinson's Disease Patients During the COVID-19 Pandemic in a Developing Country
Source: Front Neurol. 2020 Oct 27;11:582613. doi: 10.3389/fneur.2020.582613 (PMC7652791; doi:10.3389/fneur.2020.582613)
Supplement: Supplementary file 1 [file Table_1.docx]

**Supplementary Table 1: Patient’s satisfaction, set-up and quality of service scores of virtual visits**

| **No** | **Patient questionnaire** | **Median/ mean*** | **Range** |
| --- | --- | --- | --- |
| **1** | Age | 56 (10.51) | |
| **2** | Highest level of education | See table 1 | |
| **3** | Provider | Physician 1: 12 visits | |
|  |  | Physician 2: 7 visits | |
| **4** | I was able to successfully connect to my telemedicine visit and completed a visit with my neurologist. | Yes; 19 (100%), No;0 | |
| **5** | I found setting up for the visit understandable and easy. | 8 | 5 -10 |
| **6** | How long did you spend setting up for your telemedicine visit (in minutes)? This would include any time you spent preparing your computer hardware or software or internet services prior to the visit. | 12.9* (±14.3) | 1 - 60 |
| **7** | How do you feel the time commitment for your telemedicine visit compared to a face-to-face visit with your neurologist? | 9 | 6 - 10 |
| **8** | The clinical care I received during the telemedicine visit was ______ than a face-to - face visit. | 8 | 4 - 10 |
| **9** | I felt I had the doctor’s full attention during the telemedicine visit. | 10 | 5 - 10 |
| **10** | I felt my doctor could see and hear me well. | 10 | 7 - 10 |
| **11** | I felt I could see and hear my doctor well | 10 | 8 - 10 |
| **12** | I felt my body was safe during the telemedicine exam. | 10 | 9 - 10 |
| **13** | I felt my privacy was safe during the telemedicine visit. | 10 | 9 - 10 |
| **14** | Were you able to establish a personal connection to your specialist during the visit? How does it compare to an in-person visit? | [Yes, I established more of a connection] | 0 |
|  |  | [Yes I established the same level] | 4 (21%) |
|  |  | [Yes, but less of a connection] | 12 (63.1%) |
|  |  | [No, I did not establish a connection] | 3 (15.9%) |
| **15** | How satisfied were you with the specialist’s ability to provide recommendations to improve your quality of life? | [Very Satisfied] | 15 (78.9%) |
|  |  | [Satisfied] | 3 (15.9%) |
|  |  | [Neutral] | 1 (5.2%) |
|  |  | [Unsatisfied] | 0 |
|  |  | [Very Unsatisfied] | 0 |
| **16** | Do you feel that a service like this, which would allow patients to receive care from a specialist via web-based videoconferencing, would be valuable? | 19(100%) | |
| **17** | I am pleased with the outcome of my telemedicine visit. | 10 | 9 - 10 |
| **18** | If given the option, I would favor having a future telemedicine appointment. | 10 | 8 - 10 |
| **19** | If given the option, I would like my future appointments by telemedicine with my neurologist whenever feasible. | 10 | 4 - 10 |
| **20** | How likely are you to recommend telemedicine to another patient with the same or similar health condition? | [Very likely]  [Likely]  [neutral]  [unlikely]  [very unlikely] | 17 (89.5%)  2 (10.5 %)  0  0  0 |

**Supplementary Table 2: Physician’s satisfaction, set-up and quality of service scores of virtual visits**

| **No** | **Physician questionnaire** | **Median/ mean*** | **Range** |
| --- | --- | --- | --- |
| **1** | Name | Physician 1: 12 visits  Physician 2: 7 visits | |
| **2** | Date | 1 -15 June 2020 | |
| **3** | Time of virtual visit | 40 minutes appointment | |
| **4** | I was able to successfully connect to my telemedicine visit via Epic Schedule and completed a visit with my patient | Yes; 19 (100%), No;0 | |
| **5** | I found setting up for the visit understandable and easy. | 10 | 10 - 10 |
| **6** | How long did you spend setting up for your telemedicine visit (min) | 6 * (±3.55) | 2 - 10 |
| **7** | I felt the telemedicine appointment was ____ in quality overall as an office visit. | 7 | 4 - 9 |
| **8** | I felt the patient was well-focused and undistracted during the telemedicine visit. | 10 | 6 - 10 |
| **9** | I felt I could communicate well during the telemedicine visit. | 9 | 5 - 10 |
| **10** | I felt I could see and hear my patient well. | 8 | 5 - 10 |
| **11** | I felt my patient’s body was safe during all task performed during the exam | 10 | 9 - 10 |
| **12** | I felt my patient’s privacy was safe during the telemedicine visit. | 10 | 6 - 10 |
| **13** | I felt the decisions I made with my patient would have been DIFFERENT if the appointment had occurred in my office. | 1 | 1 - 5 |
| **14** | I am pleased with the outcome of this telemedicine visit. | 9 | 7 - 10 |
| **15** | If given the option I would favor having a future telemedicine appointment with my patient. | 9 | 5 - 10 |
| **16** | If given the option, I would prefer future appointment via telemedicine whenever Possible. | 8 | 7 - 9 |
